# Supplementary material for: Volatilome Analysis in Prostate Cancer by Electronic Nose: A Pilot Monocentric Study
Source: Cancers (Basel). 2022 Jun 14;14(12):2927. doi: 10.3390/cancers14122927 (PMC9220860; doi:10.3390/cancers14122927)
Supplement: Supplementary file 1 [file cancers-14-02927-s001.zip › cancers-1726066-supplementary.pdf]

Supplementary Materials: Volatilome Analysis in Prostate Cancer by Electronic Nose: A Pilot Monocentric Study

Alessio Filianoti, Manuela Costantini, Alfredo Maria Bove, Umberto Anceschi, Aldo Brassetti, Mariaconsiglia Ferriero, Riccardo Mastroianni, Leonardo Misuraca, Gabriele Tuderti, Gennaro Ciliberto and Giuseppe Simone

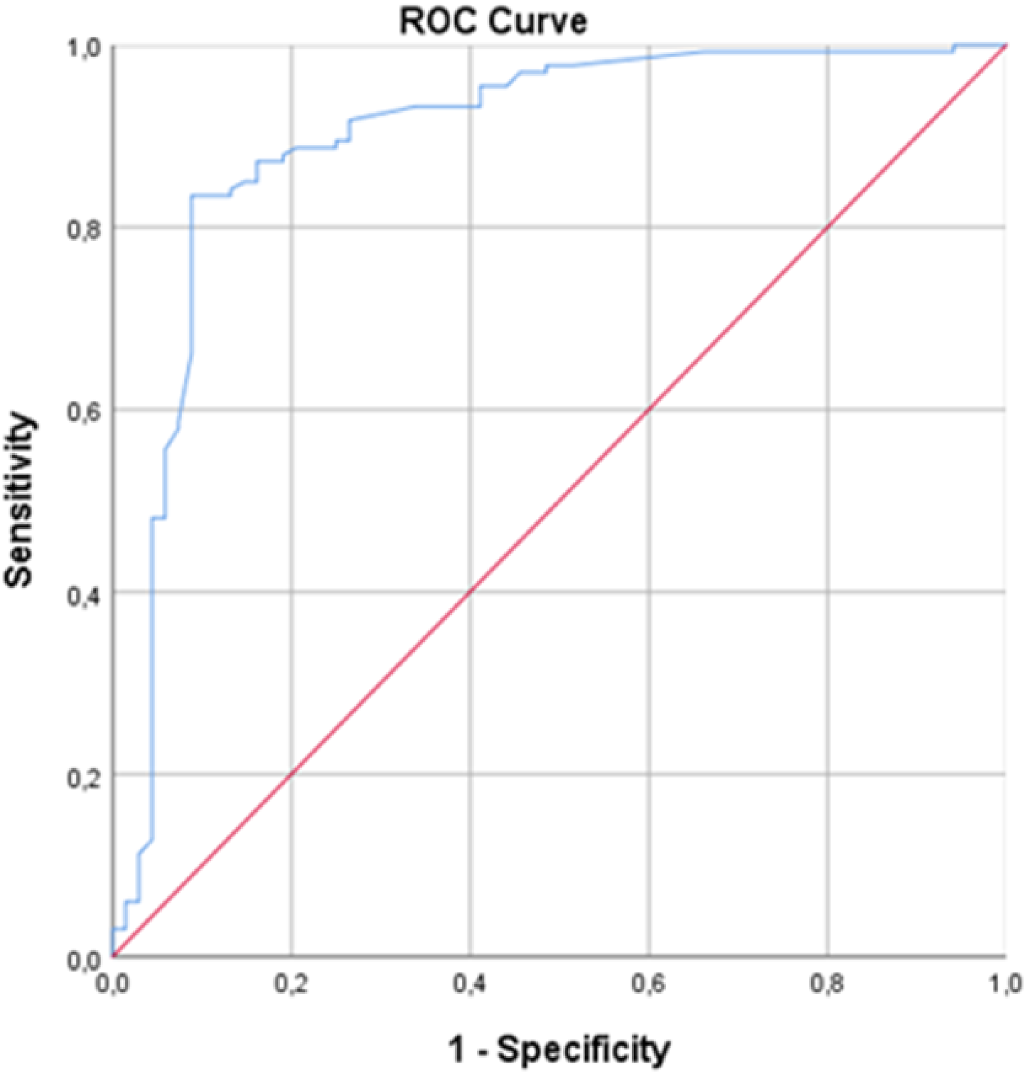

Figure S1. Psa ROC Curve analysis. At ROC analysis, discrimination accuracy (area under the curve [AUC]) was 0.89..
